# Randomized phase 2 study of perampanel for sporadic amyotrophic lateral sclerosis

**DOI:** 10.1007/s00415-021-10670-y

**Published:** 2021-06-30

**Authors:** Hitoshi Aizawa, Haruhisa Kato, Koji Oba, Takuya Kawahara, Yoshihiko Okubo, Tomoko Saito, Makiko Naito, Makoto Urushitani, Akira Tamaoka, Kiyotaka Nakamagoe, Kazuhiro Ishii, Takashi Kanda, Masahisa Katsuno, Naoki Atsuta, Yasushi Maeda, Makiko Nagai, Kazutoshi Nishiyama, Hiroyuki Ishiura, Tatsushi Toda, Akihiro Kawata, Koji Abe, Ichiro Yabe, Ikuko Takahashi-Iwata, Hidenao Sasaki, Hitoshi Warita, Masashi Aoki, Gen Sobue, Hidehiro Mizusawa, Yutaka Matsuyama, Tomohiro Haga, Shin Kwak

**Affiliations:** 1 Department of Neurology, Tokyo Medical University, Tokyo, Japan; 2 Department of Biostatics, School of Public Health, Graduate School of Medicine, The University of Tokyo, Tokyo, Japan; 3 Central Coordinating Unit, Clinical Research Support Center, The University of Tokyo Hospital, Tokyo, Japan; 4 Department of Neurology, Shiga University of Medical Science, Otsu, Japan; 5 Division of Clinical Medicine, Department of Neurology, Faculty of Medicine, University of Tsukuba, Tsukuba, Ibaraki Japan; 6 Department of Neurology and Clinical Neuroscience, Yamaguchi University Graduate School of Medicine, Ube, Japan; 7 Department of Neurology, Nagoya University, Nagoya, Japan; 8 Department of Neurology, National Hospital Organization Kumamoto Saishun Medical Center, Kumamoto, Japan; 9 Department of Neurology, Kitasato University School of Medicine, Sagamihara, Japan; 10 Department of Neurology, University of Tokyo, Tokyo, Japan; 11 Department of Neurology, Tokyo Metropolitan Neurological Hospital, Tokyo, Japan; 12 Department of Neurology, University of Okayama, Okayama, Japan; 13 Department of Neurology, Faculty of Medicine and Graduate School of Medicine, Hokkaido University, Sapporo, Japan; 14 Department of Neurology, Tohoku University Hospital, Sendai, Japan; 15 National Center of Neurology and Psychiatry, Tokyo, Japan

**Keywords:** Perampanel, Sporadic amyotrophic lateral sclerosis, AMPA receptor, Efficacy, Safety

## Abstract

**Objective:**

To evaluate the efficacy and safety of perampanel in patients with sporadic amyotrophic lateral sclerosis (SALS).

**Methods:**

This randomized, double-blind, placebo-controlled, multicenter, phase 2 clinical study was conducted at 12 sites. Patients with probable or definite ALS as defined by revised El Escorial criteria were enrolled. Sixty-six patients were randomly assigned (1:1:1) to receive placebo, 4 mg perampanel, or 8 mg perampanel daily for 48 weeks. Adverse events (AEs) were recorded throughout the trial period. The primary efficacy outcome was the change in Amyotrophic Lateral Sclerosis Rating Scale-Revised (ALSFRS-R) score after 48 weeks of treatment.

**Results:**

One patient withdrew before starting the treatment. Of 65 patients included, 18 of 22 patients randomized to placebo (82%), 14 of 22 patients randomized to 4 mg perampanel (64%), and 7 of 21 patients randomized to 8 mg perampanel (33%) completed the trial. There was a significant difference in the change of ALSFRS-R scores [− 8.4 (95% CI − 13.9 to − 2.9); *p* = 0.015] between the placebo and the perampanel 8 mg group, primarily due to worsening of the bulbar subscore in the perampanel 8 mg group. Serious AEs were more frequent in the perampanel 8 mg group than in the placebo group (*p* = 0.0483).

**Conclusions:**

Perampanel was associated with a significant decline in ALSFRS-R score and was linked to worsening of the bulbar subscore in the 8 mg group.

**Supplementary Information:**

The online version contains supplementary material available at 10.1007/s00415-021-10670-y.

## Introduction

Amyotrophic lateral sclerosis (ALS) is a fatal motor neuron disease characterized by degeneration of upper and lower motor neurons in the CNS. Its onset features progressive focal weakness and atrophy, spreading to involve most skeletal muscles, resulting in respiratory failure within 3–5 years of onset [1]. The incidence of ALS is approximately 1–2 per 100,000, and the prevalence is approximately 4–8 per 100,000 [2, 3]. The average age of onset is 58–60 years [4].

More than 90% of patients with ALS have no family history and are classified as sporadic ALS (SALS); the remaining < 10% are familial [4], usually autosomal dominant. At least 25 genes have been associated with half of familial ALS cases and a small proportion of SALS patients [5]. Recent developments in identifying culprit genes have revealed several molecular pathways [6], but there is no effective therapy. Riluzole and edaravone can modify the course of the disease but are not curative [7, 8].

The α-amino-3-hydroxy-5-methyl-4-isoxazole propionic acid (AMPA) receptor has been reported to play a pivotal role in chronic excitotoxicity of motor neurons [9–11]. Talampanel, a non-competitive AMPA receptor blocker with a half-life of 3 to 4 h [12], showed no clinical benefit in a 9-month randomized placebo-controlled trial on patients with ALS, not restricted to sporadic ALS (SALS) [13]. Perampanel, another selective non-competitive AMPA receptor antagonist, has a longer mean terminal half-life of 105 h [14], suggesting it may cause longer term blocking of AMPA receptors. Perampanel has been reported to improve physical activity and neuropathology in SALS model mice with the gene for the RNA editing enzyme adenosine deaminase acting on RNA2 (ADAR2) conditionally knocked out in the motor neurons (ADAR2^flox/flox^/VAChT.Cre-Fast or AR2) [15, 16] and presenting with a progressive ALS phenotype with abnormal inclusions of a 43-kDa Tar DNA-binding protein in the motor neurons, similar to that of SALS [16, 17]. ADAR2 regulates Ca^2+^ influx through AMPA receptors via adenosine-to-inosine conversion at the glutamine/arginine (Q/R) site of glutamate receptor-2 (GluA2) mRNA [18], making ADAR2 a key factor in acquired Ca^2+^ resistance in motor neurons. Disease-specific and site-selective ADAR2 activity on the GluA2 Q/R site is reduced in SALS motor neurons [19–21].

Based on the results of a study with perampanel in AR2 mice [15], we performed a randomized, double-blind, placebo-controlled, multicenter phase 2 clinical trial to evaluate the efficacy and safety of perampanel at daily doses of 4 mg and 8 mg, compared with placebo, in SALS patients.

## Methods

### Participants

Eligibility criteria for registration included age 40–78 years, “clinically definite ALS”, “clinically probable ALS”, or “clinically probable-laboratory supported ALS” as specified in the revised El Escorial Airlie House diagnostic criteria [22]; a maximum sum of the three respiratory items of the ALS functional rating scale-revised (ALSFRS-R) scores of 12 points; ≤ 2 years from disease onset; and the ability to visit the study site for outpatient treatment. None of the patients had a family history of ALS. Inclusion criteria after the observation period were: the ALSFRS-R score during the observation period must have decreased by 2–5 points; patients had not initiated riluzole therapy during the observation period; patients had not undergone riluzole dose escalation or resumed administration of riluzole therapy after previous dose reduction or discontinuation; and patients had not newly initiated edaravone therapy during the observation period. Exclusion criteria were tracheostomy; noninvasive positive pressure ventilation; % forced vital capacity (%FVC) < 80%; progressive bulbar palsy type; cognitive impairment; severe renal, hepatic, cardiovascular, or hematological disease; malignancy; pregnancy or the possibility of becoming pregnant; participation in another clinical study within 12 weeks before starting the observation period; and prior or current perampanel therapy.

### Study design, randomization, and blinding

The trial was conducted at 12 sites from April 2017 to January 2020. It included a 12-week observation period, followed by a randomized, double-blind, placebo-controlled, three-arm, 48-week trial period. This study followed the Consolidated Standards of Reporting Trials (CONSORT) reporting guidelines.

The patients, whose ALSFRS-R scores had decreased by 2–5 points during the 12-week observation period, were randomly assigned in a 1:1:1 ratio into three groups: one group receiving placebo, one receiving 4 mg/day of perampanel, and one receiving 8 mg/day of perampanel. Randomization was done via a computerized interactive system with the following minimization factors: change in ALSFRS-R score during the observation period (− 2 or − 3 vs*.* − 4 or − 5), sex (male vs. female), age (42–64 vs. 65–78), and the use of riluzole or edaravone (yes vs. no). After randomization of study drugs, the randomization manager created and sealed randomization codes for individual unblinding to be used in emergency situations. Participants, their families, investigators, site staff, steering committee members, and anyone involved in outcome assessments were all masked to randomization codes.

Following completion of the 12-week observation period, the patients started study treatment with perampanel 2 mg or placebo tablet once a day. The number of tablets administered per day was increased over 4 weeks to 4 placebo tablets/day in the placebo arm, 2 placebo tablets and 2 perampanel 2 mg tablets/day in the 4 mg arm, and 4 perampanel 2 mg tablets/day in the 8 mg arm. The full doses were administered daily for 44 weeks.

ALSFRS-R score, manual muscle testing (MMT) grading scale [23], and %FVC measurements were scheduled at screening and at day 0, 4 weeks, every 12 weeks, and at the followup visit. The tested muscles were as follows: bilateral biceps brachii, triceps brachii, quadriceps femoris, and hamstrings. Risk factors for suicidal behavior evaluated by the Columbia Suicide Severity Rating Scale [24] were reported at screening, and following every visit. The placebo and active tablets (Eisai Co., Ltd., Ibaraki, Japan) were quality-assured, packaged, labeled, stored at room temperature, and distributed to each study site.

### Outcomes

The primary efficacy outcome was the change in ALSFRS-R score from baseline to week 48 after randomization. The major secondary efficacy outcomes were ALSFRS-R total score (the sum of all 12 items), the ALSFRS-R score for each item, the change in ALSFRS-R score for each item, MMT grading scale [23], %FVC at 4 weeks and then every 12 weeks, and time to death or disease progression after starting the treatments. Disease progression was defined as an event as follows: inability to walk without assistance, loss of function of both upper limbs, tracheostomy, respirator use, tube feeding, or death.

Safety outcomes were determined by site investigators reporting adverse events (AEs), vital sign measurements, body weight, laboratory tests, and risk factors for suicidal behavior. AEs were reviewed by all investigators.

### Statistical analysis

The sample size in this study for each group was calculated referring to the results of an edaravone study [25]. That phase 3 study, comparing edaravone with placebo in ALS patients, reported a change of − 5.0 ± 5.3 (mean ± SD) in ALSFRS-R in the edaravone arm (*n* = 68) and − 7.5 ± 5.4 in the placebo arm (*n* = 66) (*p* = 0.0013) [25]. This study was designed to detect a dose–response relationship in changes in ALSFRS-R based on the hypothesis that perampanel would demonstrate benefits on a level similar to that of edaravone (a 3 ± 5-point maximum difference). A sample size of at least 20 patients was needed for each treatment group to have 70% power to detect a dose–response relationship with a one-sided significance level of 5% in the ALSFRS-R scores among the three groups at 48 weeks.

For the primary efficacy analysis, ALSFRS-R scores were analyzed for all patients in the intention-to-treat population. The means of changes from baseline values were calculated and analyzed using a restricted maximum likelihood (REML)-based repeated measures approach combined with the Newton Raphson algorithm. The analytical model included fixed categorical effects of treatment, visit, and treatment-by-visit interaction [26]. Akaike Information Criteria were used to choose the working correlation structures for modeling the within‐patient errors among an unstructured covariance structure, autoregressive (1) structure, and a compound symmetry structure. The Kenward–Roger approximation was used to estimate denominator degrees of freedom. The least square means of change from baseline in ALSFRS-R score during double-blind treatment and its 95% CIs were estimated and compared among the three groups. The contrast coefficients corresponding to dose–response relationships (linear dose–response relationship, dose–response curve peaking at 4 mg, dose–response curve demonstrating efficacy only at 8 mg, and dose–response curve demonstrating efficacy only at 4 mg) were tested at a one-sided significance level of 5%. The contrast test with the smallest p value was selected as the true dose–response relationship among the three groups. As a sensitivity analysis for the primary analysis, analysis of covariance was also performed for the primary endpoint after the missing data were imputed by last observation carried forward methods. Secondary endpoints were analyzed similarly through the linear mixed-effects model for repeated measures. Time to disease progression after starting the treatments, were plotted using the Kaplan–Meier method and compared among groups using log-rank tests stratified by the minimization factors. A trajectory analysis [27] was performed for exploratory analysis to identify any subgroups appearing over the trajectory of ALSFRS-R scores from baseline to 48 weeks.

All analyses, except for the primary analysis, were two-sided with a *p* value < 0.05 considered statistically significant. Analyses were performed with SAS software, version 9.4 (SAS Institute, Cary, NC, USA). PROC TRAJ macro was used for the trajectory analysis (http://www.andrew.cmu.edu/user/bjones/) [28]. This study is registered with ClinicalTrials.gov, number NCT03019419.

## Results

### Patients

The intention-to-treat-population included 65 patients. The baseline demographics and clinical characteristics were similar among the three groups (Table 1). Approximately 75% of the patients were diagnosed as having “clinically probable ALS”, or “clinically probable-laboratory supported ALS”. Most of the patients were being treated with riluzole (88%) and/or edaravone (80%).

**Table 1:** Demographic and baseline clinical characteristics of the patients^a^

	Placebo	Perampanel 4 mg	Perampanel 8 mg
Patients, *n*	22	22	21^b^
Male, *n* (%)	15 (68)	14 (64)	12(57)
Age, mean (SD), year	62.6 (9.4)	61.6 (9.8)	61.7 (9.3)
Diagnosis^c^			
Clinically definite ALS, *n* (%)	5 (22.7)	7 (31.8)	6 (28.6)
Clinically probable ALS, *n* (%)	10 (45.5)	11 (50.0)	8 (38.1)
Clinically probable ALS, *n* (%)	10 (45.5)	11 (50.0)	8 (38.1)
Clinically probable laboratory-supported ALS, *n* (%)	7 (31.8)	4 (18.2)	7 (33.3)
Duration of disease (yr)	1.28 (0.46)	0.97 (0.45)	1.06 (0.53)
Body weight (Kg)	60.8 (10.0)	58.7 (9.0)	56.4 (9.3)
ALSFRS-R score change during the observation period			
− 5 to − 4, *n* (%)	7 (32)	6 (27)	6 (29)
− 3 to − 2, *n* (%)	15 (68)	16 (73)	15 (71)
ALSFRS-R score at baseline, mean (SD)	39.5 (2.9)	40.3 (3.4)	39.9 (2.6)
Therapy at baseline			
Riluzole, *n* (%)	2 (9)	3 (14)	3 (14)
Edaravone, *n* (%)	1 (5)	2 (9)	0 (0)
Both riluzole and edaravone, *n* (%)	18 (82)	15 (68)	16 (76)
None, *n* (%)	1 (5)	2 (9)	2 (10)
MMT grading at baseline			
Biceps brachii (R), mean (SD)	3.8 (1.2)	3.7 (1.0)	4.0 (1.0)
Biceps brachii (L), mean (SD)	3.9 (1.1)	4.0 (1.2)	4.0 (0.8)
Triceps brachii (R), mean (SD)	4.2 (0.8)	4.2 (0.9)	4.1 (0.9)
Triceps brachii (L), mean (SD)	4.3 (0.8)	4.6 (0.9)	4.2 (1.0)
Quadriceps femoris (R), mean (SD)	4.6 (0.8)	4.6 (0.8)	4.9 (0.4)
Quadriceps femoris (L), mean (SD)	4.7 (0.9)	4.9 (0.5)	4.8 (0.4)
Hamstrings (R), mean (SD)	4.6 (0.6)	4.5 (0.8)	4.5 (0.7)
Hamstrings (L), mean (SD)	4.5 (0.9)	4.7 (0.6)	4.4 (0.7)
Sum score of MMT grading, mean (SD)	34.6 (3.4)	35.1 (3.4)	34.9 (3.2)
%FVC at baseline, mean (SD)	98.9 (17.2)	90.4 (16.9)	92.9 (14.3)

Abbreviations: *ALS* amyotrophic lateral sclerosis; *ALSFRS-R* ALS functional rating scale-revised; *MMT* manual muscle testing; *%FVC* percent-predicted forced vital capacity; *R* right side; *L* left side

^a^ There were no significant differences (*p* < 0.05) among the study groups

^b^ One patient withdrew from the trial without starting the medication

^c^ Progressive bulbar type was excluded in this trial

**Fig. 1:**
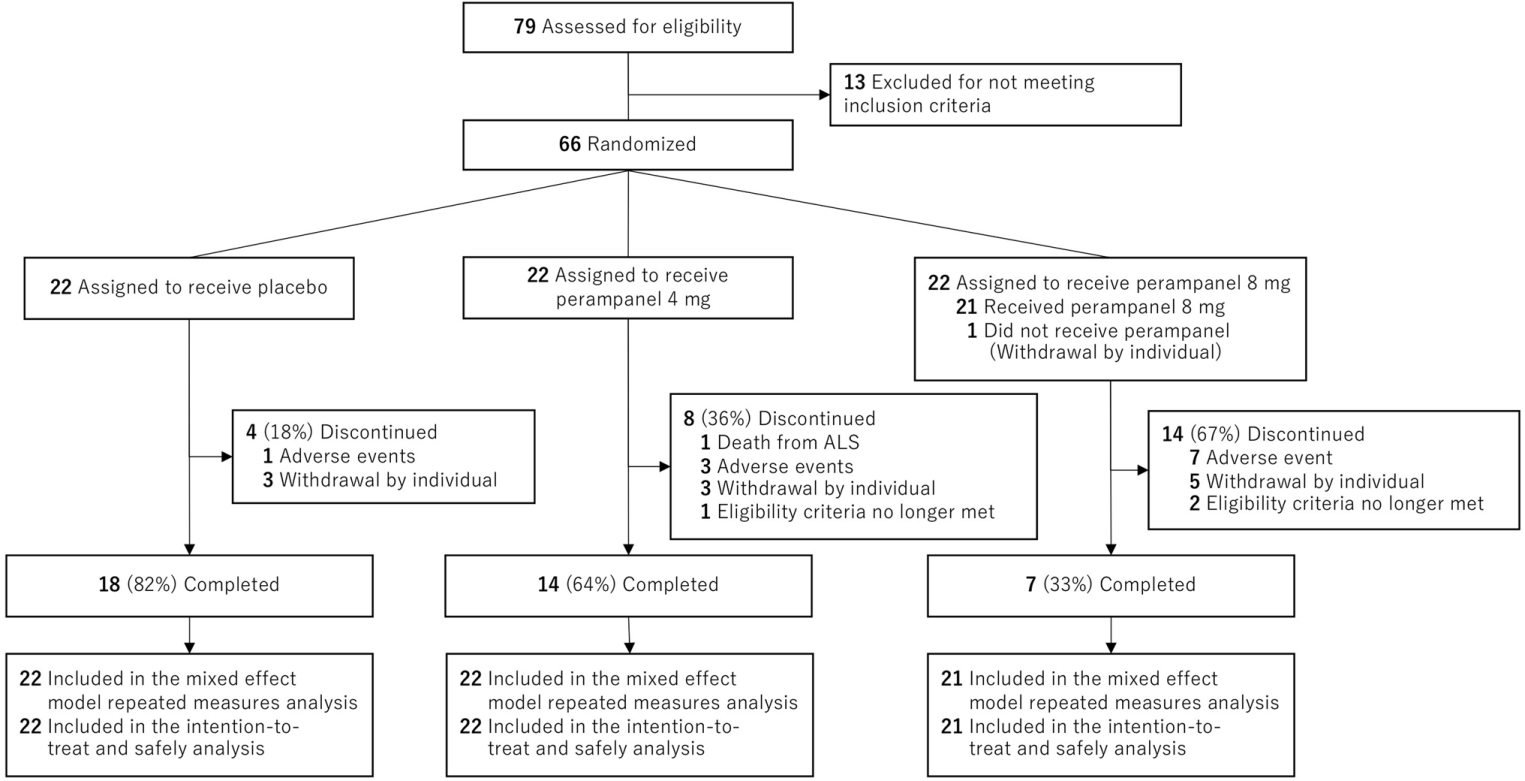
Enrolment and randomization of patients. Randomization was stratified according to change in ALS functional rating scale-revised (ALSFRS-R) score during the observation period (− 2 or − 3 vs*.* − 4 or − 5), sex (male vs*.* female), age (42 to 64 vs. 65 to 78), and the use of riluzole or edaravone (yes vs. no)

The discontinuation rate of the trial increased in a dose-dependent manner, with 18 of 22 patients (82%) in the placebo group, 14 of 22 patients (64%) in the 4 mg perampanel group, and 7 of 21 patients (33%) in the 8 mg perampanel group completing the 48-week trial. Of the 26 patients who did not complete the trial, one patient died of ALS, 11 had AEs, 11 declined to participate further, and 3 did not meet continuation criteria (one patient: impossible to take tablet orally; 2 patients: unable to visit the study site) (Fig. 1).

### Efficacy

**Fig. 2:**
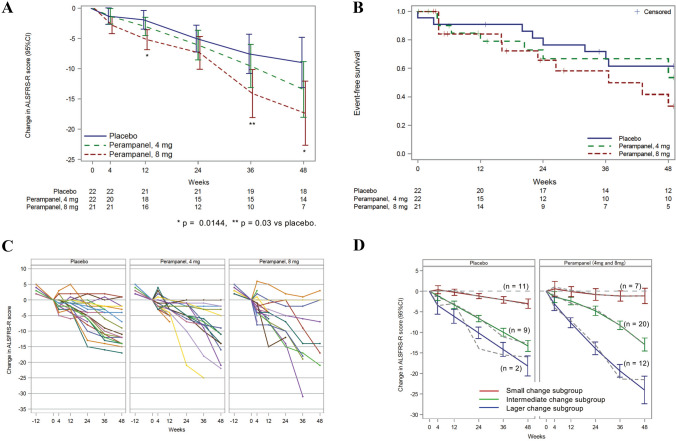
Changes in ALSFRS-R score and Kaplan–Meier curve. **A** Mean change in ALSFRS-R score over time from the baseline in the placebo group, 4 mg perampanel group, and 8 mg perampanel groups. Error bars represent standard deviations (SDs) for the mean values at each timepoint. **B** Changes in ALSFRS-R score over time from the baseline for each ALS patient. There were wide variations in the changes of ALSFRS-R scores over the treatment period within each group. **C** The Kaplan–Meier curve for time to death or disease progression. There was no significant difference in dropout rate due to disease progression (inability to walk without assistance, loss of function of both upper limbs, tracheostomy, respirator use, tube feeding, or death) among the three groups. Some patients were followed for 48 weeks after disease progression (6, 4, and 2 patients in placebo, 4 mg, and 8 mg groups, respectively). (D) Changes in ALSFRS-R score suggested by trajectory analysis. Cases were classified into three subgroups; a subgroup with small changes (red line; *n* = 11 in the placebo group and *n* = 7 in the perampanel group), a subgroup with intermediate changes (green line; *n* = 9 in the placebo group and *n* = 20 in the perampanel group), and a subgroup with large changes (blue line; *n* = 2 in the placebo group and *n* = 12 in the perampanel group). Solid lines are estimated curves from the trajectory analysis and dotted lines are the mean of the measured values. Error bars represent 95% CIs for the mean values at each timepoint

Evaluation of the dose–response relationship did not reach statistical significance (one-sided *p* = 0.463 for a dose–response curve demonstrating efficacy only at 4 mg, Table S-1). The estimated mean change of ALSFRS-R total scores from baseline to week 48 was − 9.0 in the placebo group (*n* = 18), − 13.4 in the 4 mg perampanel group (*n* = 14), and − 17.4 in the 8 mg perampanel group (*n* = 7) (*p* = 0.01 for the comparison between the placebo group and the 8 mg perampanel group) (Table 2). The changes in the ALSFRS-R score from the baseline were larger in the 8 mg perampanel groups compared to the placebo group at 12, 36, and 48 weeks (Fig. 2a; Table 3). The change of bulbar subscores in the 8 mg perampanel group were significantly higher than those of the control group (*p* = 0.0206); there were no significant differences in upper limb, lower limb, or respiratory subscores among placebo and perampanel groups (Table 2). The Kaplan–Meier curve for time to disease progression did not differ among groups (log-rank; *p* = 0.276, Fig. 2b). In addition, the ALS Milano-Torino Staging (MiToS) system [29], which correlates well with functional stage and QOL in ALS, was retrospectively evaluated using the ALSFRS-R score. MiToS staging at week 48 was not significantly different among the three groups (data not shown).

**Table 2:** Primary and secondary outcomes^a^

	Placebo (95% CI)	Perampanel 4 mg (95% CI)	Perampanel 8 mg (95% CI)	Perampanel 4 mg vs. Placebo (95% CI)	*p* value	Perampanel 8 mg vs. Placebo (95% CI)	*p* value
Primary outcome							
Changes in ALSFRS-R score at 48 weeks from baseline	− 9.0 (− 13.1 to − 4.8)	− 13.4 (− 18.0 to − 8.8)	− 17.4 (− 22.7 to − 12.1)	− 4.5 (− 10.6 to 1.6)	0.1476	− 8.4 (− 15.0 to − 1.8)	0.0145
Number of patients at 48 weeks	18	14	7				
Secondary outcome							
Changes in ALSFRS − R score at 48 weeks from baseline^b^	− 6.9 (− 10.9 to − 2.9)	− 9.4 (− 13.2 to − 5.7)	− 8.2 (− 12.3 to − 4.1)	− 2.5 (− 6.9 to 1.9)	0.2574	− 1.3 (− 5.6 to 3.0)	0.5519
Number of patients at 48 weeks	22	20	21				
Bulbar subscore^c^	− 0.8 (− 2.2 to 0.6)	− 2.2 (− 3.7 to − 0.6)	− 3.5 (− 5.3 to − 1.7)	− 1.4 (− 3.5 to 0.7)	0.1760	− 2.7 (− 5.0 to − 0.4)	0.0206
Upper limbs subscore^d^	− 4.3 (− 5.8 to − 2.9)	− 4.2 (− 5.8 to − 2.6)	− 5.5 (− 7.4 to − 3.6)	0.1 (− 1.9 to 2.2)	0.8872	− 1.2 (− 3.5 to 1.1)	0.3028
Lower limbs subscore^e^	− 3.1 (− 4.5 to − 1.7)	− 4.4 (− 5.9 to − 2.9)	− 4.3 (− 6.1 to − 2.6)	− 1.2 (− 3.2 to 0.7)	0.2046	− 1.2 (− 3.4 to 0.9)	0.2583
Respiratory subscore^f^	− 0.3 (− 1.3 to 0.8)	− 1.6 (− 2.8 to − 0.4)	− 1.6 (− 3.1 to − 0.1)	− 1.3 (− 2.9 to 0.3)	0.1029	− 1.4 (− 3.2 to 0.5)	0.1375
Total ALSFRS-R score at 48 weeks^g^	30.7 (26.6 to 34.8)	27.2 (22.8 to 31.7)	23.6 (18.4 to 28.9)	− 3.5 (− 9.3 to 2.4)	0.2351	− 7.1 (− 13.5 to − 0.7)	0.0315
Changes in MMT grading at 48 weeks from baseline^g^							
Biceps brachii (R)	− 1.4 (− 1.9 to − 0.8)	− 1.3 (− 1.9 to − 0.7)	− 0.8 (− 1.5 to − 0.1)	0.1 (− 0.7 to 0.9)	0.7924	0.6 (− 0.3 to 1.4)	0.2006
Biceps brachii (L)	− 1.2 (− 1.7 to − 0.7)	− 0.9 (− 1.4 to − 0.3)	− 1.4 (− 2.2 to 0.7)	0.3 (− 0.4 to 1.1)	0.3962	− 0.3 (− 1.1 to 0.6)	0.5425
Triceps brachii (R)	− 0.9 (− 1.4 to − 0.3)	− 0.9 (− 1.5 to − 0.2)	− 0.7 (− 1.5 to 0.1)	0.0 (− 0.8 to 0.8)	0.9879	0.1 (− 0.8 to 1.1)	0.7710
Triceps brachii (L)	− 1.1 (− 1.6 to − 0.6)	− 0.8 (− 1.4 to − 0.3)	− 0.8 (− 1.5 to − 0.0)	0.3 (− 0.4 to 1.0)	0.3792	0.4 (− 0.4 to 1.2)	0.3517
Quadriceps femoris (R)	− 0.7 (− 1.3 to − 0.1)	− 0.6 (− 1.3 to 0.0)	− 1.2 (− 2.0 to − 0.4)	0.1 (− 0.8 to 0.9)	0.8432	− 0.5 (− 1.5 to 0.5)	0.3019
Quadriceps femoris (L)	− 0.8 (− 1.4 to − 0.3)	− 0.5 (− 1.2 to 0.1)	− 0.8 (− 1.6 to 0.0)	0.3 (− 0.5 to 1.1)	0.483	0.0 (− 0.9 to 1.0)	0.9802
Hamstrings (R)	− 1.0 (− 1.5 to − 0.5)	− 0.5 (− 1.0 to 0.1)	− 0.2 (− 1.0 to 0.5)	0.6 (− 0.2 to 1.3)	0.1387	0.8 (− 0.1 to 1.6)	0.0852
Hamstrings (L)	− 1.0 (− 1.5 to − 0.5)	− 0.5 (− 1.0 to 0.0)	− 0.1 (− 0.7 to 0.6)	0.5 (− 0.2 to 1.2)	0.1417	0.9 (0.1 to 1.7)	0.0216
Sum score of MMT grading	− 8.3 (− 11.3 to − 5.4)	− 6.1 (− 9.4 to − 2.8)	− 6.5 (− 10.6 to − 2.4)	2.2 (− 2.1 to 6.6)	0.3098	1.8 (− 3.1 to 6.8)	0.4574
Changes in %FVC at 48 weeks from baseline	− 24.9 (− 35.3 to − 14.6)	− 31.4 (− 43.2 to − 19.6)	− 33.9 (− 48.5 to − 19.4)	− 6.4 (− 21.9 to 9.0)	0.4027	− 9.0 (− 26.5 to 8.5)	0.3058

*ALS *amyotrophic lateral sclerosis, *ALSFRS-R *ALS functional rating scale-revised, *MMT *manual muscle testing

^a^Analyses are based on data from the intention-to-treat population for all end points

^b^Covariance analysis was also used for the changes in ALSFRS-R score at 48 weeks from baseline

^c^Bulbar subscore is sum of scores for items 1, 2, and 3 (speech, salivation, and swallowing) in ALSFRS-R

^d^Upper limb subscore is sum of scores for items 4, 5, and 6 (handwriting cutting food and handling utensils, and dressing and hygiene) in ALSFRS-R

^e^Lower limb subscore is sum of scores for items 7, 8, and 9 (turning in bed and adjusting bed clothes, walking, and climbing stairs) in ALSFRS-R

^f^Respiratory subscore is sum of scores for items 10, 11, and 12 (dyspnea, orthopnea, and respiratory insufficiency) in ALSFRS-R

^g^Number of patients at each week were the same as those in the total ALSFRS-R score

**Table 3:** Other secondary outcomes

	Placebo (95% CI)	Perampanel 4 mg (95% CI)	Perampanel 8 mg (95% CI)	Perampanel 4 mg vs. Placebo (95% CI)	*p* value	Perampanel 8 mg vs. Placebo (95% CI)	*p* value
Changes in ALSFRS-R score							
At 4 weeks from baseline, n	− 1.3 (− 2.7 to –0.1), 22	− 1.3 (− 2.6 to − 0.0), 20	− 2.8 (− 4.2 to − 1.4), 21	− 0.0 (− 1.5 to 1.5)	0.9899	− 1.5 (− 3.0 to 0.0)	0.0526
At 12 weeks from baseline, n	− 1.9 (− 3.5 to − 0.4), 21	− 3.0 (− 4.5 to − 1.5), 18	− 5.2 (− 6.9 to − 3.5), 16	− 1.1 (− 3.0 to 0.8)	0.2495	− 3.3 (− 5.2 to − 1.4)	0.0012
At 24 weeks from baseline, n	− 5.0 (− 7.2 to − 2.8), 21	− 6.1 (− 8.5 to − 3.6), 15	− 7.4 (− 10.1 to − 4.7), 12	− 1.1 (− 4.2 to 2.1)	0.4953	− 2.4 (− 5.7 to 0.9)	0.1532
At 36 weeks from baseline, n	− 7.6 (− 10.8 to − 4.3), 19	− 9.6 (− 13.1 to − 6.0), 14	− 14.1 (− 18.1 to − 10.1), 10	− 2.0 (− 6.7 to 2.7)	0.3951	− 6.6 (− 11.6 to − 1.6)	0.0112
At 48 weeks from baseline, n	− 9.0 (− 13.1 to − 4.8), 18	− 13.4 (− 18.0 to − 8.8), 14	− 17.4 (− 22.7 to − 12.1), 7	− 4.5 (− 10.6 to 1.6)	0.1476	− 8.4 (− 15.0 to − 1.8)	0.0145
Total ALSFRS-R score^a^							
At 4 weeks	38.3 (36.3 to 40.3)	38.8 (36.9 to 40.7)	37.1 (35.1 to 39.1)	0.5 (− 1.7 to 2.7)	0.6722	− 1.3 (− 3.4 to 0.9)	0.2484
At 12 weeks	37.7 (35.7 to 39.7)	37.1 (35.1 to 39.0)	34.8 (32.6 to 36.9)	− 0.6 (− 2.9 to 1.7)	0.5813	− 2.9 (− 5.2 to − 0.6)	0.0142
At 24 weeks	34.6 (32.1 to 37.1)	34.0 (31.4 to 36.7)	32.7 (29.7 to 35.6)	− 0.6 (− 3.9 to 2.8)	0.7325	− 1.9 (− 5.4 to 1.5)	0.2665
At 36 weeks	32.1 (28.7 to 35.4)	30.9 (27.3 to 34.5)	26.3 (22.2 to 30.3)	− 1.2 (− 5.9 to 3.5)	0.6150	− 5.8 (− 10.8 to − 0.9)	0.0225
At 48 weeks	30.7 (26.6 to 34.8)	27.2 (22.8 to 31.7)	23.6 (18.4 to 28.9)	− 3.5 (− 9.3 to 2.4)	0.2351	− 7.1 (− 13.5 to − 0.7)	0.0315

*ALS *amyotrophic lateral sclerosis, *ALSFRS-R *ALS functional rating scale-revised

Analyses are based on date from the intention-to-treat population for all end points

^a^Numbers of patients at each week are same as those in the changes in ALSFRS-R scores

In the subgroup analysis (changes in ALSFRS-R scores during the observation period, sex, age, and the use of riluzole or edaravone), the patients with small but significant decreases (− 3 to − 2) in ALSFRS-R score during the observation period (*p* = 0.0467), and those taking riluzole and/or edaravone (*p* = 0.0374), had a significant decrease in the score if they went on to take 8 mg perampanel, suggesting high-dose perampanel may promote disease progression in patients taking riluzole and/or edaravone. (Tables S-2 and S-3).

The changes in ALSFRS-R scores varied more than expected within each group, and some patients in each group experienced changes in ALSFRS-R scores ranging from 3 to − 5 after randomization through the 48 weeks (Fig. 2c). Because of the wide variations in the changes of ALSFRS-R scores over the treatment period, trajectory analysis was performed in the placebo and perampanel groups. In the trajectory analysis, the perampanel groups were merged because of their high dropout rates. The results suggested three different patterns of progression (Fig. 2d). It is of note that the pattern of small changes (red line) in the perampanel group was nonprogressive as compared to the corresponding pattern in the placebo group that showed a steady decline. On the other hand, the pattern of large changes (blue line) in the perampanel groups showed a faster decline than that in the placebo group. To elucidate the differences in clinical features of subsets according to the trajectory analysis, we retrospectively compared the changes in ALSFRS-R scores during the observation period and the baseline characteristics between nonprogressive and progressive subsets on trajectory analysis (i.e. the small change subset vs. the intermediate and large change subset) (Table S-4). In the placebo group, the small change subset (nonprogressive) showed a higher total ALSFRS-R score at baseline (*p* = 0.0407) and smaller changes in score during the observation period (*p* = 0.0105) than the progressive subset (Table S-4). In the perampanel group, the small change subset (nonprogressive) had lower subscores in the lower limbs in ALSFRS-R at baseline (*p* = 0.0081) and longer duration from disease onset (*p* = 0.0007) than the progressive subset (Table S-4).

**Fig. 3:**
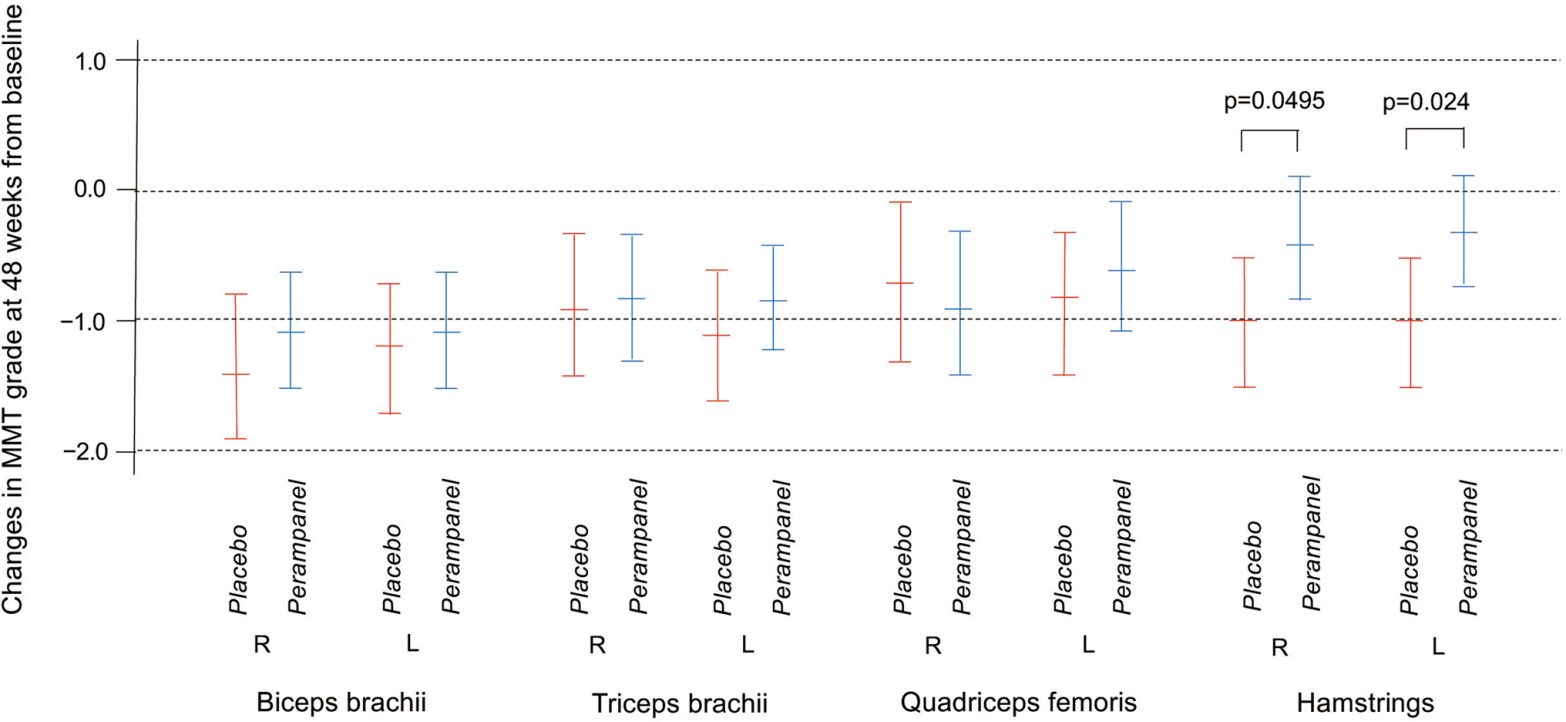
Changes in MMT grades at 48 weeks from baseline in placebo and perampanel groups. Data are mean (95% CI). *MMT *manual muscle testing

Changes in MMT grading from baseline to week 48 showed no significant difference among the three groups except that the deterioration in MMT grading of the left hamstring muscles was significantly smaller in the 8 mg perampanel group than that in the placebo group (Table 2). When comparing the placebo group to the perampanel group (4 mg and 8 mg), the change in MMT grading of the hamstrings on both sides was significantly smaller in the perampanel group than in the placebo group (Fig. 3). There was no statistically significant difference in the mean decline of %FVC after 48 weeks among the three groups (Table 2).

### Safety

The percentage of patients with reported serious AEs was 13.6% in the placebo group, 27.3% in the 4 mg perampanel group, and 47.6% in the 8 mg perampanel group (Table 4), more frequent in the 8 mg perampanel group than in the placebo group (*p* = 0.0483). One patient in the 4 mg perampanel group died of ALS. Treatment-related AEs such as dizziness and excessive sleepiness were reported more frequently in the 8 mg perampanel group (16 events) and 4 mg perampanel group (11 events) than in the placebo group (one event). There were no significant differences in the results of laboratory tests, vital signs, ECGs, or chest X-rays among the three groups.

**Table 4:** Adverse events^a^

	Placebo	Perampanel 4 mg	Perampanel 8 mg
Total participants, *n*	22	22	21
Adverse effects, *n* (%)			
Any adverse event^b^	22 (100)	20 (91)	21 (100)
Any serious adverse events^c^	3 (14)	6 (27)	10 (48)
Death	0	1 (5)	0
Dysphagia requiring gastrostomy	2 (9)	4 (18)	6 (29)
Respiratory failure	2 (9)	2 (9)	0
Gastric disturbance	0	0	3 (14)
Infection	1 (5)	1 (5)	1 (5)
Fracture/injury	0	1 (5)	1 (5)
Anger/hallucination	0	0	2 (10)
DIC	0	0	1 (5)
Malignancy	0	0	1 (5)
Any adverse events affecting > 5% in any group, *n* (%)			
Gait disturbance	6 (27)	6 (27)	8 (38)
Fall/injury	1 (5)	2 (9)	2 (10)
Dysphagia	1 (5)	1 (5)	5 (24)
Gastrostomy	2 (9)	4 (18)	5 (24)
Dizziness	3 (14)	8 (36)	8 (38)
Vertigo	3 (14)	3 (14)	2 (10)
Somnolence	0	4 (18)	8 (38)
Anxiety	0	0	2 (10)
Sleep disturbance	3 (14)	2 (9)	2 (10)
Dyspnea	3 (14)	3 (14)	1 (5)
Upper respiratory infection	8 (36)	3 (14)	6 (29)
Nausea	0	1 (5)	2 (10)
Liver dysfunction	5 (24)	2 (9)	1 (5)
Constipation	8 (36)	2 (9)	6 (29)
Urinary tract infection	4 (18)	1 (5)	4 (19)
Urinary disturbance	2 (9)	0	0
Joint pain	2 (9)	2 (9)	2 (10)
Skin ulcer	2 (9)	0	1 (5)
Tooth extraction	0	2 (9)	0
Anemia	0	0	3 (14)

*DIC *disseminated intravascular coagulation

^a^Definitions of adverse events are provided in the Trial Protocol in Supplement 1

^b^No significant difference in the frequency of any adverse event was reported. When multiple adverse events occurred, they always occurred in one patient

^c^Serious adverse events were more frequent in the 8 mg perampanel group than in the placebo group (*p* = 0.0483). A serious adverse event is an adverse event that: (1) results in death; (2) is life-threatening; (3) requires inpatient hospitalization or prolongation of existing hospitalization; (4) results in persistent or significant disability/incapacity; or (5) is another medically important condition

## Discussion

Based on the results, the major finding of this trial is that perampanel did not show efficacy in SALS. On the contrary, the results suggest a potential detrimental effect. However, this result may be influenced by the high percentage of dropouts and the study may have not been powered enough to demonstrate this effect. The main reason for worsening of ALSFRS-R scores in the 8 mg perampanel group compared to the placebo group was a significant decrease in the bulbar subscore, since there were no significant differences in limb and respiratory subscores between the placebo and perampanel groups. The results suggest that a higher dose of perampanel may promote, rather than prevent, deterioration of bulbar function in SALS patients. This has been reported as an adverse event in patients with epilepsy [30]. The subgroup analysis suggested that high-dose perampanel may promote disease progression in patients taking riluzole and/or edaravone, although the precise mechanism of interaction among these drugs is unknown.

The rate of ALSFRS-R score decline in each patient varied widely in the perampanel groups as well as in the placebo group, and trajectory analysis suggested three progressive pattern models, made up of small, intermediate, and large changes. The subset of small change of trajectory curves in the placebo group, about one-third of the patients, had slower progression throughout the observation and treatment periods than the other two placebo group subsets. The subset of small changes in trajectory curves in the perampanel groups had longer disease duration with more weakness in the lower limbs at baseline compared to the subset of intermediate and large changes. In these patients, MMT grade was virtually nonprogressive throughout the 48-week treatment period in the majority of limb muscles, and was better preserved in some muscles compared to the subset of small changes in the placebo group.

Glutamate excitotoxicity via AMPA receptors is a plausible hypothesis in motoneuron degeneration in ALS. Topiramate not only reduces the membrane depolarization by AMPA/kainate receptors but also has multiple effects, such as blocking voltage-dependent sodium channels, enhancing GABA (A) receptors, and down-regulating NMDA receptor activity; however, it could not prevent ALS progression, probably because of its low selective antagonism to AMPA receptors or the high doses used [31]. Ceftriaxone, a beta-lactam antibiotic, showed negative results in preventing ALS deterioration in spite of its increased EAAT2 activity [32], partially due to the lack of a pharmacodynamic marker of ceftriaxone to upregulate glutamate transporter in patients with ALS. Talampanel, a non-competitive AMPA receptor blocker with a relatively short half-life [12], was not beneficial for ALS [13]. Perampanel, a non-competitive AMPA receptor blocker with a longer half-life, showed no clinical benefit using the ALSFRS-R score in this study*.* Perampanel also has effects on molecules other than AMPA receptors, including modulation of voltage-gated sodium channel and M-type potassium currents [33], and regulation of several kinases [34]. Perampanel did not exert beneficial effects on the lower motor neurons and exerted negative effects on other neurons in the CNS, particularly at the 8 mg dose, possibly due to modulation of molecules other than AMPA receptors [33, 34]. Its non-AMPA antagonistic function may have dominated in the subsets of SALS patients displaying disease acceleration. A higher percentage of participants with a lower rate of ALSFRS-R score progression in the placebo group may have masked any beneficial effects. Considering the hypothesis that ADAR2 regulates Ca^2+^ influx through AMPA receptors [9–11, 18–21], further study of drugs that have competitive AMPA antagonism or upregulate ADAR2 activity is warranted.

Dizziness and excessive sleepiness were frequent AEs with perampanel as previously reported [35], although these events disappeared with lowering the dose. High doses of perampanel are not recommended because serious AEs were dose-related.

This trial had several limitations. First, this phase 2 trial was performed in a relatively small population. Second, of 66 patients, only 39 patients (59%) were able to complete the trial. The main reasons for withdrawal were disease progression and AEs related to perampanel, particularly in the high-dose group.

## Conclusion

This trial did not support a neuroprotective effect of perampanel but suggests instead a potential detrimental effect at high doses. Since there was a high percentage of patients discontinuing the treatment, as well as considerable variability of change in the ALSFRS-R score at 48 weeks from baseline among the individuals in each group, including virtual non-progression in some participants in the perampanel groups, the clinical benefit of perampanel in SALS patients needs further study.

## Supplementary Information

Below is the link to the electronic supplementary material.

Supplementary file1 (DOCX 20 kb)

Supplementary file2 (DOCX 56 kb)

## Data Availability

The data for the analyses described in this paper, study protocol, informed consent forms, and statistical analysis plan are available from H.A. (haizawa@tokyo-med.ac.jp) by request, with a signed date access agreement.
