# Supplementary material for: Randomized phase 2 study of perampanel for sporadic amyotrophic lateral sclerosis
Source: J Neurol. 2021 Jun 30;269(2):885–96. doi: 10.1007/s00415-021-10670-y (PMC8782807; doi:10.1007/s00415-021-10670-y)
Supplement: Supplementary file 1 — Supplementary file1 (DOCX 20 kb) [file 415_2021_10670_MOESM1_ESM.docx]

**Supplemental Table. MiToS score**^a^

|  | Placebo  (95% CI) | Perampanel 4 mg  (95% CI) | Perampanel 8 mg  (95% CI) | Perampanel 4 mg vs. Placebo  (95% CI) | *P*- value | Perampanel 8 mg vs. Placebo  (95% CI) | *P*- value |
| --- | --- | --- | --- | --- | --- | --- | --- |
| Changes in MiToS score from baseline |  |  |  |  |  |  |  |
| at 4 weeks | 0.3 (0.0 to 0.5) | 0.1 (−0.1 to 0.3) | 0.4 (0.1 to 0.6) | −0.2 (−0.4 to 0.1) | 0.1874 | 0.1 (−0.1 to 0.4) | 0.413 |
| at 12 weeks | 0.4 (0.1 to 0.6) | 0.2 (0 to 0.5) | 0.7 (0.4 to 1.0) | −0.1 (−0.5 to 0.2) | 0.3834 | 0.3 (0 to 0.7) | 0.0379 |
| at 24 weeks | 0.7 (0.3 to 1.0) | 0.5 (0.1 to 0.9) | 1.0 (0.6 to 1.4) | −0.2 (−0.6 to 0.3) | 0.501 | 0.3 (−0.2 to 0.8) | 0.1918 |
| at 36 weeks | 0.9 (0.5 to 1.4) | 0.8 (0.3 to 1.3) | 1.7 (1.1 to 2.2) | −0.1 (−0.7 to 0.5) | 0.793 | 0.3 (0.1 to 1.5) | 0.0269 |
| at 48 weeks | 1.1 (0.6 to 1.6) | 1.2 (0.6 to 1.7) | 1.9 (1.2 to 2.6) | 0 (−0.7 to 0.8) | 0.9288 | 0.8 (-0.1 to 1.6) | 0.0643 |

Abbreviations: MiToS = Milano-Torino staging, CI = confidence intervals

^a^Analyses are based on the data from the intention-to-treat population for all end points.
