# Supplementary material for: Randomized phase 2 study of perampanel for sporadic amyotrophic lateral sclerosis
Source: J Neurol. 2021 Jun 30;269(2):885–96. doi: 10.1007/s00415-021-10670-y (PMC8782807; doi:10.1007/s00415-021-10670-y)
Supplement: Supplementary file 2 — Supplementary file2 (DOCX 56 kb) [file 415_2021_10670_MOESM2_ESM.docx]

Supplementary Information

Journal of Neurology

Randomized phase 2 study of perampanel for sporadic amyotrophic lateral sclerosis

Hitoshi Aizawa^1^ · Haruhisa Kato^1^ · Koji Oba^2^ · Takuya Kawahara^3^ · Yoshihiko Okubo^1^ · Tomoko Saito^1^ · Makiko Naito^1^ · Makoto Urushitani^4^ · Akira Tamaoka^5^ · Kiyotaka Nakamagoe^5^ · Kazuhiro Ishii^5^ · Takashi Kanda^6^ · Masahisa Katsuno^7^ · Naoki Atsuta^7^ · Yasushi Maeda^8^ · Makiko Nagai^9^ · Kazutoshi Nishiyama^9^ · Hiroyuki Ishiura^10^ · Tatsushi Toda^10^ · Akihiro Kawata^11^ · Koji Abe^12^ · Ichiro Yabe^13^ · Ikuko Takahashi-Iwata^13^ · Hidenao Sasaki^13^ · Hitoshi Warita^14^ · Masashi Aoki^14^ · Gen Sobue^7^ · Hidehiro Mizusawa^15^ · Yutaka Matsuyama^2^ · Tomohiro Haga^3^ · Shin Kwak^1^

Correspondence: Hitoshi Aizawa (DRCID ID: 0000-0002-5395-695X)

[haizawa@tokyo-med.ac.jp](mailto:haizawa@tokyo-med.ac.jp)

1. Department of Neurology, Tokyo Medical University, Tokyo, Japan
2. Department of Biostatics, School of Public Health, Graduate School of Medicine, The University of Tokyo, Tokyo, Japan
3. Central Coordinating Unit, Clinical Research Support Center, The University of Tokyo Hospital, Tokyo, Japan
4. Department of Neurology, Shiga University of Medical Science, Otsu, Japan
5. Department of Neurology, Division of Clinical Medicine, Faculty of Medicine, University of Tsukuba, Tsukuba, Ibaraki, Japan
6. Department of Neurology and Clinical Neuroscience, Yamaguchi University Graduate School of Medicine, Ube, Japan
7. Department of Neurology, Nagoya University, Nagoya, Japan
8. Department of Neurology, National Hospital Organization Kumamoto Saishun Medical Center, Kumamoto, Japan
9. Department of Neurology, Kitasato University School of Medicine, Sagamihara, Japan
10. Department of Neurology, University of Tokyo, Tokyo, Japan
11. Department of Neurology, Tokyo Metropolitan Neurological Hospital, Tokyo, Japan
12. Department of Neurology, University of Okayama, Okayama, Japan
13. Department of Neurology, Faculty of Medicine and Graduate School of Medicine, Hokkaido University, Sapporo, Japan
14. Department of Neurology, Tohoku University Hospital, Sendai, Japan
15. National Center of Neurology and Psychiatry, Tokyo, Japan

Table S-1. Evaluation of dose-response relationship (primary analysis)

Table S-2. Analyses of Treatment Effects on ALS subgroups (Placebo group vs. Perampanel groups)

Table S-3. Analyses of Treatment Effects on ALS subgroups

Table S-4. Retrospective Analyses based on Treatment Effects on ALS subgroups according to Trajectory Analysis

Appendix S-1. CONSORT 2020 checklist of information

**Table S-1. Evaluation of dose-response relationship (primary analysis)**

| **Fitted Contrast** | **Contrast coefficient** | **Estimate** | **SE** | **t-value** | **p-value** ^a^ |
| --- | --- | --- | --- | --- | --- |
| Linear dose-response relationship | [−1, 0, 1] | −8.4 | 3.3 | −2.5512 | 0.9928 |
| Dose-response curve peaking at 4 mg | [−2, 1, 1] | −12.9 | 5.3 | −2.4281 | 0.9900 |
| Dose-response curve demonstrating efficacy only at 8 mg | [−1, −1, 2] | −12.3 | 6.0 | −2.0493 | 0.9768 |
| Dose-response curve demonstrating efficacy only at 4 mg | [−1, 2, −1] | 0.5 | 5.6 | 0.0947 | 0.4625 |

Abbreviation: SE = standard error.

^a^ One-sided *p*-value.

**Table S-2. Analyses of Treatment Effects on ALS subgroups^a^**

|  | **Placebo**  (95% CI) | **Perampanel 4 mg**  (95% CI) | **Perampanel 8 mg**  (95% CI) | **Perampanel 4 mg vs. Placebo**  (95% CI) | ***p* Value** | **Perampanel 8 mg vs. Placebo**  (95% CI) | ***p* Value** |
| --- | --- | --- | --- | --- | --- | --- | --- |
| Changes in ALSFRS-R score at 48 weeks from baseline |  |  |  |  |  |  |  |
| Sex |  |  |  |  |  |  |  |
| Male | −9.2 (−13.8 to −4.6) | −15.4 (−21.0 to −9.8) | −13.8 (−20.3 to −7.4) | −6.2 (−13.3 to 1.0) | 0.0872 | −4.6 (−12.4 to 3.1) | 0.2309 |
| Patients at 48 weeks | 12 | 7 | 5 |  |  |  |  |
| Female | −8.8 (−18.0 to 0.4) | −10.7 (−19.9 to −1.5) | −22.1 (−32.4 to −11.8) | −1.9 (−14.8 to 11.0) | 0.7519 | −13.3 (−27.1 to 0.5) | 0.0574 |
| Patients at 48 weeks | 6 | 7 | 2 |  |  |  |  |
| Age |  |  |  |  |  |  |  |
| <65 y | −8.9 (−13·4 to −4.3) | −14.0 (−19.1 to −9.0) | −12.9 (−18.2 to −7.6) | −5.2 (−11.8 to 1.5) | 0.1195 | −4.0 (−10.9 to 2.8) | 0.2327 |
| patients at 48 weeks | 10 | 8 | 5 |  |  |  |  |
| ≥65 y | −8.9 (−17.5 to −0.4) | −11.6 (−20.9 to −2.4) | −30.2 (−42.7 to −17.6) | −2.7 (−15.2 to 9.8) | 0.6520 | −21.2 (−36.3 to −6.2) | 0.0081 |
| patients at 24 weeks | 8 | 6 | 2 |  |  |  |  |
| Changes in ALSFRS-R during 12 weeks of observation period |  |  |  |  |  |  |  |
| −5–−4 | −12.6 (−23.9 to −1.2) | −16.7 (−30.4 to −3.0) | −22.2 (−34.8 to −9.6) | −4.1 (−21.8 to 13.6) | 0.6004 | −9.6 (−26.6 to 7.3) | 0.2220 |
| patients at 48 weeks | 5 | 3 | 2 |  |  |  |  |
| −3–−2 | −7.0 (−12.0 to −2.1) | −11.7 (−17.0 to −6.4) | −15.4 (−22.0 to −8.7) | −4.6 (−11.8 to 2.5) | 0.1944 | −8.3 (−16.5 to −0.1) | 0.0467 |
| patients at 48 weeks | 13 | 11 | 5 |  |  |  |  |
| Edaravone or riluzole |  |  |  |  |  |  |  |
| Yes | −8.9(−12.9 to −4.8) | −14.8(−19.6 to −10.0) | −16.1(−21.6 to −10.6) | −5.9 (−12.2 to 0.4) | 0.0646 | −7.2 (−14.0 to −0.4) | 0.0374 |
| patients at 48 weeks | 18 | 12 | 7 |  |  |  |  |

Abbreviations: ALS = amyotrophic lateral sclerosis; ALSFRS-R = ALS functional rating scale-revised.

^a^ Analyses are based on date from the intention-to-treat population for all end points.

**Table S-3. Analyses of Treatment Effects on ALS subgroups (Placebo group vs. Perampanel groups)^a^**

|  | **Placebo**  (95% CI) | **Perampanel**  **4 mg + 8 mg**  (95% CI) | **Perampanel**  **4 mg + 8 mg vs. Placebo**  (95% CI) | **p Value** |
| --- | --- | --- | --- | --- |
| Changes in ALSFRS-R score at 48 weeks from baseline |  |  |  |  |
| Sex |  |  |  |  |
| Male | −9.1 (−13.6 to −4.6) | −14.6 (−18.8 to −10.4) | −5.5 (−11.5 to 0.5) | 0.0706 |
| patients at 48 weeks | 12 | 12 |  |  |
| Female | −9.6 (−21.2 to 1.9) | −18.3 (−26.6 to −10.0) | −8.7 (−22.8 to 5.4) | 0.2017 |
| patients at 48 weeks | 6 | 9 |  |  |
| Age |  |  |  |  |
| <65 y | −8.9 (−13.5 to −4.4) | −13.9 (−17.6 to −10.1) | −4.9 (−10.6 to 0.8) | 0.0888 |
| patients at 48 weeks | 10 | 13 |  |  |
| ≥65 y | −8.8 (−17.5 to 0.0) | −18.4 (−26.1 to −10.6) | −9·6 (−21.1 to 1.9) | 0.0944 |
| patients at 24 weeks | 8 | 8 |  |  |
| Changes in ALSFRS-R during 12 weeks of observation period |  |  |  |  |
| −5–−4 | −11.5 (−21.7 to −1.4) | −19.0 (−27.3 to −10.7) | −7.5 (−20.4 to 5.5) | 0.2199 |
| patients at 48 weeks | 5 | 5 |  |  |
| −3–−2 | −7.1 (−12.3 to −2.0) | −13.6 (−18.0 to −9.3) | −6.5 (−13.1 to 0.1) | 0.0540 |
| patients at 48 weeks | 13 | 16 |  |  |
| Edaravone or riluzole |  |  |  |  |
| Yes | −8.9 (−13.0 to −4.8) | −15.7 (−19.3 to −12.0) | −6.8 (−12.3 to −1.3) | 0.0163 |
| patients at 48 weeks | 18 | 19 |  |  |

Abbreviations: ALS = amyotrophic lateral sclerosis; ALSFRS-R = ALS functional rating scale-revised; MMT = manual muscle testing; R = right side; L = left side; %FVC = percent-predicted forced vital capacity.

^a^ Analyses are based on date from the intention-to-treat population for all endpoints.

**Table e-4. Retrospective Analyses based on Treatment Effects on ALS subgroups according to Trajectory Analysis ^a^**

|  | Placebo | Placebo | Perampanel | Perampanel |
| --- | --- | --- | --- | --- |
|  | Small changes in ALSFRS-R score  (N=11)  (95% CI) | Intermediate-to- large changes in ALSFRS-R score  (N=11)  (95% CI) | Small changes in ALSFRS-R score  (N=9)  (95% CI) | Intermediate-to- large changes in ALSFRS-R score  (N=32)  (95% CI) |
| **ALSFRS-R score at baseline** |  |  |  |  |
| Total score, mean (SD) | 40.7 (2.7) | 38.3 (2.6) | 38.9 (3.0) | 40.4 (3.0) |
| *p* value compared to small changes |  | 0.0407 |  | 0.1851 |
| Bulbar subscore, mean (SD) | 11.5 (0.9) | 11.5 (0.7) | 11.9 (0.3) | 11.3 (1.1) |
| *p* value compared to small changes |  | 0.7976 |  | 0.1384 |
| Upper limbs subscore, mean (SD) | 8.7 (1.5) | 7.7 (2.2) | 8.6 (2.7) | 8.2 (1.3) |
| *p* value compared to small changes |  | 0.2256 |  | 0.6072 |
| Lower limbs subscore, mean (SD) | 8.5 (2.4) | 7.1 (2.5) | 6.4 (1.8) | 8.9 (2.5) |
| *p* value compared to small changes |  | 0.2059 |  | 0.0081 |
| Respiratory subscore, mean (SD) | 12.0 (0.0) | 12.0 (0.0) | 12.0 (0.0) | 12.0 (0.2) |
| *p* value compared to small changes |  | ― |  | 0.6022 |
| **Baseline characteristics** |  |  |  |  |
| Age, mean (SD) | 63.2 (10.2) | 62.0 (9.0) | 65.4 (10.0) | 60.5 (9.3) |
| *p* value compared to small changes |  | 0.7763 |  | 0.1772 |
| Duration of ALS, mean (SD), year | 1.23 (0.49) | 1.33 (0.43) | 1.48 (0.38) | 0.89 (0.44) |
| *p* value compared to small changes |  | 0.6306 |  | 0.0007 |
| Changes in ALSFRS-R during 12 weeks |  |  |  |  |
| of observation period, mean (SD) | −2.4 (0.7) | −3.5 (1.2) | −2.8 (1.0) | −2.9 (1.0) |
| *p* value compared to small changes |  | 0.0105 |  | 0.6835 |

Abbreviations: ALS = amyotrophic lateral sclerosis; ALSFRS-R = ALS functional rating scale-revised; MMT = manual muscle testing; R = right side; L = left side; %FVC = percent-predicted forced vital capacity.

^a^ Analyses are based on date from the intention-to-treat population for all end points.

Appendix S-1.


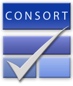
 CONSORT 2010 checklist of information to include when reporting a randomised trial*

| Section/Topic | Item No | Checklist item | Reported on page No |
| --- | --- | --- | --- |
| Title and abstract | | | |
|  | 1a | Identification as a randomised trial in the title | 1 |
|  | 1b | Structured summary of trial design, methods, results, and conclusions (for specific guidance see CONSORT for abstracts) | 1 |
| Introduction | | | |
| Background and objectives | 2a | Scientific background and explanation of rationale | 2 |
|  | 2b | Specific objectives or hypotheses | 2 |
| Methods | | | |
| Trial design | 3a | Description of trial design (such as parallel, factorial) including allocation ratio | 3 |
|  | 3b | Important changes to methods after trial commencement (such as eligibility criteria), with reasons | N/A |
| Participants | 4a | Eligibility criteria for participants | 3 |
|  | 4b | Settings and locations where the data were collected | 3 |
| Interventions | 5 | The interventions for each group with sufficient details to allow replication, including how and when they were actually administered | 3, 4 |
| Outcomes | 6a | Completely defined pre-specified primary and secondary outcome measures, including how and when they were assessed | 4 |
|  | 6b | Any changes to trial outcomes after the trial commenced, with reasons | N/A |
| Sample size | 7a | How sample size was determined | 4, 5 |
|  | 7b | When applicable, explanation of any interim analyses and stopping guidelines | N/A |
| Randomisation: |  |  |  |
| Sequence generation | 8a | Method used to generate the random allocation sequence | 4 |
|  | 8b | Type of randomisation; details of any restriction (such as blocking and block size) | 4 |
| Allocation concealment mechanism | 9 | Mechanism used to implement the random allocation sequence (such as sequentially numbered containers), describing any steps taken to conceal the sequence until interventions were assigned | 4 |
| Implementation | 10 | Who generated the random allocation sequence, who enrolled participants, and who assigned participants to interventions | 4 |
| Blinding | 11a | If done, who was blinded after assignment to interventions (for example, participants, care providers, those assessing outcomes) and how | 4 |
|  | 11b | If relevant, description of the similarity of interventions | 4 |
| Statistical methods | 12a | Statistical methods used to compare groups for primary and secondary outcomes | 4, 5 |
|  | 12b | Methods for additional analyses, such as subgroup analyses and adjusted analyses | 4, 5 |
| Results | | | |
| Participant flow (a diagram is strongly recommended) | 13a | For each group, the numbers of participants who were randomly assigned, received intended treatment, and were analysed for the primary outcome | 6  Figure 1 |
|  | 13b | For each group, losses and exclusions after randomisation, together with reasons | Figure 1 |
| Recruitment | 14a | Dates defining the periods of recruitment and follow-up | 6 |
|  | 14b | Why the trial ended or was stopped | N/A |
| Baseline data | 15 | A table showing baseline demographic and clinical characteristics for each group | Table 1 |
| Numbers analysed | 16 | For each group, number of participants (denominator) included in each analysis and whether the analysis was by original assigned groups | Figure1, Table 2 |
| Outcomes and estimation | 17a | For each primary and secondary outcome, results for each group, and the estimated effect size and its precision (such as 95% confidence interval) | 6  Table 2 |
|  | 17b | For binary outcomes, presentation of both absolute and relative effect sizes is recommended | N/A |
| Ancillary analyses | 18 | Results of any other analyses performed, including subgroup analyses and adjusted analyses, distinguishing pre-specified from exploratory | Table 2, 3, and Fig 1, 2, 3 |
| Harms | 19 | All important harms or unintended effects in each group (for specific guidance see CONSORT for harms) | 7, Table 4 |
| Discussion | | | |
| Limitations | 20 | Trial limitations, addressing sources of potential bias, imprecision, and, if relevant, multiplicity of analyses | 7, 8 |
| Generalisability | 21 | Generalisability (external validity, applicability) of the trial findings | 8 |
| Interpretation | 22 | Interpretation consistent with results, balancing benefits and harms, and considering other relevant evidence | 8, 9 |
| Other information | | |  |
| Registration | 23 | Registration number and name of trial registry | 5 |
| Protocol | 24 | Where the full trial protocol can be accessed, if available |  |
| Funding | 25 | Sources of funding and other support (such as supply of drugs), role of funders | 9 |

*We strongly recommend reading this statement in conjunction with the CONSORT 2010 Explanation and Elaboration for important clarifications on all the items. If relevant, we also recommend reading CONSORT extensions for cluster randomised trials, non-inferiority and equivalence trials, non-pharmacological treatments, herbal interventions, and pragmatic trials. Additional extensions are forthcoming: for those and for up to date references relevant to this checklist, see [www.consort-statement.org](http://www.consort-statement.org).
